# Community pharmacists' involvement in smoking cessation: familiarity and implementation of the National smoking cessation guideline in Finland

**DOI:** 10.1186/1471-2458-10-444

**Published:** 2010-07-29

**Authors:** Terhi Kurko, Kari Linden, Kirsi Pietilä, Patrick Sandström, Marja Airaksinen

**Affiliations:** 1 University of Helsinki, Faculty of Pharmacy, Division of Social Pharmacy, Viikinkaari 9C, P.O Box 56, 00014 University of Helsinki, Finland; 2 Pfizer Oy, Helsinki, Finland; 3 Pharmacy of Malmi, Helsinki, Finland; 4 The National Institute of Health and Welfare, Helsinki, Finland

## Abstract

**Background:**

Guidelines on smoking cessation (SC) emphasize healthcare cooperation and community pharmacists' involvement. This study explored the familiarity and implementation of the National SC Guideline in Finnish community pharmacies, factors relating to Guideline familiarity, implementation and provision of SC services.

**Methods:**

A nationwide mail survey was sent to a systematic, sample of community pharmacy owners and staff pharmacists (total n = 2291). Response rate was 54% (n = 1190). Factors related to the SC Guideline familiarity were assessed by bivariate and multivariate analysis.

**Results:**

Almost half (47%) of the respondents (n = 1190) were familiar with the SC Guideline and familiarity enhanced Guideline implementation. The familiarity was associated with the respondents' perceptions of their personal SC skills and knowledge (OR 3.8); of customers' value of counseling on nicotine replacement therapy (NRT) (OR 3.3); and regular use of a pocket card supporting SC counseling (OR 3.0). Pharmacists' workplaces' characteristics, such as size and geographical location were not associated with familiarity. In addition to recommending NRT, the pharmacists familiar with the Guideline used more frequently other Guideline-based SC methods, such as recommended non-pharmacological SC aids, compared to unfamiliar respondents.

**Conclusions:**

SC Guideline familiarity and implementation is crucial for community pharmacists' involvement in SC actions in addition to selling NRT products. Pharmacists can constitute a potential public health resource in SC easily accessible throughout the country.

## Background

Smoking cessation (SC) is one of the most effective ways to promote public health and reduce healthcare costs [1-5]. Health care professionals play a key role in SC [1,4,5]. To support SC, evidence-based guidelines have been established [4-7]. Despite this, SC does not routinely take place in healthcare in the recommended way [4,6-10]. Recent British and US guidelines on SC emphasize cooperation in healthcare and community pharmacists' involvement [4,5]. Correspondingly, the Finnish Current Care Guideline (later SC Guideline) published in 2002 and updated in 2006 has been developed from the multidisciplinary viewpoint by the Finnish Medical Society [7]. According to it, all healthcare professionals have specified SC responsibilities, and the local cooperation between them should facilitate SC by increasing and strengthening contacts with patients [11,12].

Some studies of the implementation process of the SC guidelines have been made [4,6,10,13]. These studies and the literature on guideline implementation provide strong evidence that changing healthcare professionals' practices requires more effort than solely disseminating the guidelines; systematic efforts are needed to promote their usage [4,13-15]. Most of these studies are focused on physicians and their practices, and few studies have explored the implementation of guidelines among other healthcare professionals [6,10,14,16-19]. Community pharmacists' role in SC has been widely studied and according to the systematic reviews conducted, their participation in SC may increase cessation rates [20-22]. Still, community pharmacists could extent their participation in SC by several alternative actions (Additional file 1, Table S1) [8,23-27]. To our knowledge this is the first study exploring the implementation of a National SC guideline among them.

The Finnish healthcare system is largely based on the public healthcare system, which is complemented by private sector services [28]. The National public health policy has had a strong emphasis on preventive services [28,29]. SC has been one of the priorities in preventive healthcare, with the focus on strong socioeconomic gradient in smoking prevalence [30]. Local SC services are coordinated by the healthcare centers [28,29].

Finnish community pharmacies are privately owned by pharmacists and highly regulated to emphasize public health goals in their operations [31]. Finnish community pharmacists have proactively developed professional services, particularly patient counseling on prescription and non-prescription medications [32]. A long-term strategic goal of the Association of Finnish Pharmacies (AFP; the association of pharmacy owners) has been to establish a network of specialized community pharmacists on major public health concerns coordinating local services for customers having asthma, diabetes or cardiovascular diseases [33]. These initiatives have been supported by authorities and there is evidence that they have been successful [32]. The Finnish medicine users value the Finnish pharmacy system and pharmacists are appreciated among the three most commonly used and reliable sources of medicines information in addition to physicians and patient information leaflets [34,35].

Finnish community pharmacists are strongly encouraged to be involved in SC by the SC Guideline [7]. The professional organizations, particularly the Association of Finnish Pharmacies, have actively supported community pharmacists' involvement in SC and the implementation of the SC Guideline, e.g., by national training campaigns and SC counseling aid materials [36].

The SC Guideline was developed by a multidisciplinary expert group and it is based on robust scientific evidence [7]. It provides background information on tobacco and smoking as a health risk. Furthermore, the Guideline introduces a wide range of interventions found effective in SC, which are applicable in various health care settings. It's recommendations are targeted to all health care professionals including community pharmacists (Additional File 2, Table S2). The Guideline is distributed nationwide by free internet access along with online education supporting its implementation.

The objective of this study was to explore the implementation of the National SC Guideline in Finnish community pharmacies, factors related to familiarity with it and its implementation, and actions taken to provide SC services.

## Methods

### Study design

The survey questionnaire was mailed to a systematic national sample of every second Finnish community pharmacist (n = 2291), including pharmacy owners (n = 292) and staff pharmacists (n = 2235) in November 2006. The sample was systematically drawn from member registers of the three national pharmacists' professional associations. At the time of the survey, their registers covered 100% of the Finnish pharmacy owners and 93% of the staff pharmacists with B.Sc. (Pharm.) and M.Sc. (Pharm.) degree. Finnish pharmacy owners belong to the Association of Finnish Pharmacies (AFP). Staff pharmacists with a B.Sc (Pharm.) belong to the Finnish Pharmacists' Association and staff pharmacists with a M.Sc. (Pharm.) degree belong to either the Finnish Pharmacists' Association (FPA) (with 369 M.Sc. members at the time of the survey) or the Finnish Pharmacists' Society (FPS) (with 379 members). Thus, the survey was sent to 185 M.Sc. members of FPA and to 190 M.Sc. members of FPS. For the statistical analysis all the received responses were combined.

After two reminders and one repeat questionnaire, we received responses from a total of 1190 community pharmacists, the final response rate being 54% (Table 1). The respondents well represented the target population according to demographic characteristics, but respondents who specialized in the treatment of asthma, diabetes or cardiovascular diseases were of a higher percentage compared to those unspecialized in those fields (Table 1).

**Table 1:** Characteristics of the respondents (n = 1190) and the target population (n = 5053).

Variable	Respondents (n = 1190)		Target population (n = 5053)		p-value**
	
	%	n	%	n	
Variables associated with the respondent pharmacist					
Professional status					0.858
B.Sc. Pharmacist (Staff pharmacist)	70	833	72	3654	
M.Sc. Pharmacist (Staff pharmacist)	18	217	16	815	
Pharmacy owner	12	140	12	584	
Total	100	1190	100	5053	
Specialization of the respondent*					<0.001
Asthma	25	272	14	706	
Diabetes	21	230	13	680	
Cardiovascular diseases	20	221	12	613	
Participation in continuing education in SC					
In-house training	48	568	na	na	
Continuing education (CE)	25	302	na	na	
In-house training by a drug company	40	477	na	na	
CE by a drug company	33	398	na	na	
Not participated	20	229	na	na	
Variables associated with the respondent's working place					
Location by province					0.790
Southern Finland	39	459	41	2087	
Western Finland	33	392	35	1773	
Eastern Finland	15	174	12	604	
Northern Finland	13	157	12	589	
Total	100	1182	100	5053	
Annual prescription volume					0.161
Less than 40 000	21	252	16	782	
40 000-80 000	38	452	34	1751	
80 001 or more	41	475	50	2520	
Total	100	1179	100	5053	
Ownership					0.295
Privately owned	94	1108	91	4589	
University owned	6	75	9	464	
Total	100	1183	100	5053	

* Based on the professional programs run by the AFP since late 1990 s to assure community pharmacies' contribution to the national public health goals. Information available on pharmacists working in privately owned community pharmacies (missing data from the two university owned pharmacies covering about 11% of the total prescription volume).

** Counted between the of respondents and the target population in relation to the background variable

na = not available

The study was conducted following scientific ethics [37]. To secure respondents anonymity, all the responses were handled in numeric form. No ethics committee's approval was required for this kind of study under the ethical guidelines of the University of Helsinki.

### Survey instrument

The survey instrument was based on the literature on guideline dissemination and implementation [6,13-16,38,39]. As the familiarity with a guideline is the first step in implementation [15], one of the key questions in our survey was designed to assess just this. The structured question was adopted from earlier studies conducted among physicians and public health nurses (Sandström P et al. 2009, unpublished) and was modified for pharmacy practice as follows: "How familiar are you with the SC Guideline?" The following options were given: "I have read the Guideline through carefully; I am familiar with its main principles; I have glanced through it; I have heard about it"; and "I am not familiar with it". For the analysis the options "I have read the Guideline carefully through" and "I am familiar with its main principles" were combined to make up a group of respondents "Familiar with the Guideline." The remainder of the respondents was categorized as "Unfamiliar with the Guideline".

According to the Finnish SC Guideline, it is the duty of the pharmacy owners to arrange for sufficient SC training for their staff pharmacists (Additional file 2, Table S2) [7]. This was measured by asking respondents to assess their participation in continuing SC education (Tables 1, 2 and 3). The SC Guideline was used as a basis for the measuring of SC actions taken at community pharmacies (Table 4). It recommends community pharmacists to ensure sufficient and rational use of SC medication and to support non-pharmacological SC [7]. This means that pharmacists are expected to assess individual customer's level of addiction to plan the treatment and its follow-up accordingly. They are also expected to recommend the use of non-pharmacological SC aids, such as written materials or internet portals. In our survey this was measured by a set of questions assessing the frequency of recommending Guideline-based pharmacological and non-pharmacological SC treatment options to the smoking pharmacy customers (Table 4).

**Table 2:** Proportion (%) of the respondents familiar with the SC Guideline (n = 525).

Variable	%	p-value*
Factors associated with the responding pharmacist		
Professional status		<0.001
B.Sc. Pharmacist (Staff pharmacist)	41	
M.Sc. Pharmacist (Staff pharmacist)	51	
Pharmacy owner	59	
Age (years)		<0.001
29 or less	35	
30-39	41	
40-49	48	
50-59	51	
60 or more	57	
Graduation date		0.020
In the 2000's	41	
In the 1990's	42	
In the 1980's	48	
In the 1970's or earlier	53	
Specialization in asthma**		<0.001
Yes	61	
No	40	
Specialization in diabetes**		0.082
Yes	40	
No	46	
Specialization in cardiovascular diseases**		0.093
Yes	50	
No	44	
Participation in continuing education supporting SC		<0.001
Yes	50	
No	23	
Perceives health care's support needed in SC		<0.001
Always or almost always	51	
Rarely	39	
Never	20	
Perceives own personal skills and knowledge adequate to support SC		<0.001
Agree	50	
No opinion	36	
Disagree	17	
Perceives customers value of NRT counseling		0.025
Agree	46	
No opinion	44	
Disagree	27	
Perceives cigarette's smoke extremely detrimental to health (Scale: 1 = strongly disagree; 10 = strongly agree)		0.024
Yes	46	
No	36	
Smoked at least 100 cigarettes up to date		0.003
Yes	52	
No	42	
Factors associated with the respondent's workplace		
Location by province		0.042
Southern Finland	47	
Eastern Finland	40	
Western Finland	42	
Northern Finland	53	
Annual prescription volume		0.276
Less than 40 000	42	
40 000-80 000	48	
80 001 or more	44	
Ownership		0.990
Privately owned	45	
University owned	45	
Dispensing counter design		<0.001
Traditional counter	37	
Sit-down counseling station	48	
Both in use	51	

SC services available in the respondent's workplace		
Individually tailored SC service		0.005
Yes	57	
No	44	
Smoking pass		0.005
Yes, used often	75	
Yes, used sometimes	61	
No	46	
SC counseling supporting pocket card in use		<0.001
Yes, used often	64	
Yes, used sometimes	48	
No	31	
In-house guideline in NRT dispensing		<0.001
Yes, written guideline available	59	
Yes, oral guideline available	56	
No	36	
Participation in local multidisciplinary SC actions		<0.001
Yes	69	
No	39	

Total (all the respondents)	47	

* Difference between the levels of each background variable.

**Based on the professional programs run by the AFP since late 1990 s to assure community pharmacies' contribution to the national public health goals. Information available on pharmacists working in privately owned community pharmacies (missing data from the two university owned pharmacies covering about 11% of the total prescription volume).

**Table 3:** Variables associated with the familiarity of SC Guideline in the multivariate analysis (logistic regression analysis)

Variable	Unadjusted		Adjusted*	
		
	OR (95% Cl)	p-value	OR (95% CI)	p-value
Factors associated with the responding pharmacist				
Specialization in asthma				
No	1.0		1.0	
Yes	2.3 (1.8-3.1)	<0.001	2.1 (1.5-3.0)	<0.001
Participation in education supporting SC counselling				
No	1.0		1.0	
Yes	3.3 (2.4-4.7)	<0.001	2.1 (1.5-3.1)	<0.001
Perceives health care's support needed in SC				
Never	1.0		1.0	
Seldom	2.6 (1.2-5.7)	0.010	2.0 (0.9-5.0)	0.104
Always or almost always	4.3 (1.9-9.4)	0.021	2.7 (1.2-6.6)	0.022
Perceives own personal skills and knowledge adequate to support SC				
No	1.0		1.0	
No opinion	2.8 (1.5-5.2)	0.001	3.5 (1.6-7.4)	0.001
Yes	5.0 (3.1-7.9)	<0.001	3.8 (2.2-6.5)	<0.001
Perceives customers value of NRT counseling				
Disagree	1.0		1.0	
No opinion	2.2 (1.0-4.7)	0.016	3.3(1.3-8.7)	0.016
Agree	2.4 (1.2-4.5)	0.044	2.3 (1.0-5.4)	0.044
Smoked at least 100 cigarettes up to date				
No	1.0		1.0	
Yes	1.5 (1.1-1.9)	0.003	1.7 (1.2-2.3)	0.001

SC services available in the respondent's workplace				
SC counseling supporting pocket card in use				
Never	1.0		1.0	
Sometimes	2.0 (1.5-2.6)	<0.001	1.7 (1.3-2.4)	0.002
Always	3.9 (2.8-5.5)	<0.001	3.0 (2.0-4.4)	<0.001
In-house guideline in NRT dispensing in use				
No	1.0		1.0	
Yes, oral	2.3 (1.7-3.1)	<0.001	1.5 (1.0-2.1)	0.030
Yes, written	2.4 (1.8-3.3)	<0.001	1.8 (1.3-2.6)	0.001
Participation in local multidisciplinary SC actions				
No	1.0		1.0	
Yes	3.5 (2.5-4.8)	<0.001	2.4 (1.6-3.5)	<0.001

* Adjusted for all the variables shown in the table. Logistic regression analysis: R^2 ^= 0.264 (Nagelkerke) and Goodness-of-fit test (Hosmer-Lemeshow): 1,312; p = 0.995. CI = Confidence Interval

**Table 4:** Implementation of the SC Guideline-based actions among the pharmacists familiar and unfamiliar with the Guideline.

SC Guideline based actions and services	Familiar with the SC Guideline (n = 512)	Unfamiliar with the SC Guideline (n = 625)	p-value*
	%	%	
Workplace involvement in local multidisciplinary SC actions			
Participation in a joint training	15	5	<0.001
Considered joint practices	12	5	<0.001
Have joint practices	5	2	<0.001
Recommends SC Guideline-based pharmacological treatment			
Nicotine gum	94	96	0.450
Nicotine patch	86	80	0.010
Nicotine inhaler	22	20	0.345
Bupropion (Zyban^®^)	15	10	0.080
Recommends SC Guideline-based non-pharmacological tools			
Own quitting decision	68	62	0.020
Written SC support material	56	41	<0.001
Participation in "Quit and Win" competition	26	23	0.242
Support of family and/or friends	17	14	0.120
Telephone-based SC support	12	8	0.090
Pharmacy's individually tailored SC service	10	6	0.006
Group therapy	9	5	0.027
Internet-based SC support	7	4	0.080
Advise to see a public health nurse	4	3	0.104
Follows at "least often" ** the 5A's Intervention with smoking customers.			
Advised to quit during the month prior the survey	12	3	<0.001
Told about how smoking effects medication during past month	10	2	<0.001
Asked about smoking during past week	9	2	<0.001
Assessed quitting date during past month	2	1	<0.001
Discusses always*** about smoking with customers who			
Self-refer to their smoking	94	91	0.101
are pregnant	45	32	<0.001
buy SC medicines	35	17	<0.001
suffer from a smoking-related disease	18	7	<0.001

* Between the groups familiar and unfamiliar with the SC Guideline

** Reclassified from the options: "always", "often", "at least with every second customer", "sometimes, never". "Often" was considered as option "often or more frequently"

*** "Always" from the original answering options: "always", " sometimes", "never"

In addition, the SC Guideline [7] expects pharmacists to be familiar with the Finnish version of the 5A's Intervention (6K's in Finnish) [4,40]. Respondents were asked about the use of the 5A's Intervention with the options: "Always; Often; With every second customer; Sometimes; Never." For the analysis, options "Always" and "Often" were reclassified as "At least often" (Table 4). The remaining options were categorized as "Not often".

### Background variables

The background variables used in this study were selected on the basis of the literature [13,15,16,38,39]. They were related to characteristics of the responding pharmacist; characteristics of his/her workplace; and SC actions that have taken place at his/her workplace (Table 2). This categorization of the background variables originates from the meta-review of Francke et al. (2008), in which guideline implementation was identified to be affected by factors related to guideline content, patient, healthcare professional and environment [15]. In addition to these variables, participation in local SC multidisciplinary teamwork was used as a background variable (Tables 2 and 3) for familiarity with the Guideline and measuring its implementation (Table 4).

All background variables for Guideline familiarity and implementation were used in their original format, except the one related to pharmacist's participation in continuing SC education (Table 1). It was reclassified as a dichotomic variable (yes/no) for the statistical analysis (Table 2). In the Likert-type statements measuring respondents' perceptions of the importance of SC and healthcare's role in it (Table 2), the options "Always" and "Almost always" were reclassified as "Always"; options "Strongly agree" and "Agree" to "Agree"; and correspondingly "Disagree" and "Strongly disagree" to "Disagree". The pharmacy's location by province was re-classified to four regions: Southern, Eastern, Western and Northern (combination of options Oulu and Lapland) Finland (Tables 1 and 2).

### Statistical analysis

The statistical analysis was based on bivariate and multivariate analysis. The statistical significance in the bivariate analysis was tested by Chi Square test (p-value of <0.05 was considered statistically significant). In the multivariate analysis, logistic regression was applied to control for a concomitant effect of the background variables selected for the analysis on the basis of the bivariate analysis. The main outcome variable measuring respondents' familiarity with the Guideline was dichotomized (1 = Respondent familiar with the Guideline; 0 = Unfamiliar). The Spearman's correlations between the background variables were calculated to avoid possible multicolinearity. Of the two variable pairs with a correlation co-efficient of 0.6 or more, (respondent's age and graduation year; and "Smoking pass in use" and "Pocket card to support SC counseling in use"), respondent's age and use of a SC pocket card were included in the regression analysis. A backward step-wise logistic regression analysis was conducted. The final model did not include interaction terms. The associations between the outcome and background variables were described by Odds Ratios calculated from the B-estimates, and p-values (Table 3). All the statistical analyses were conducted by the SPSS analytical software, version 15 (SPSS Inc., Chicago, IL).

## Results

### Familiarity with the SC Guideline

Almost half (47%) of the responding pharmacists (n = 1190) were familiar with the SC Guideline (Table 2). In bivariate analysis, the following variables related to individual pharmacists' characteristics positively influenced the familiarity with the SC Guideline: specialization in asthma; ever smoked 100 or more cigarettes; participation in continuing SC education; being a pharmacy owner; and being older (Table 2). Also the following variables reflecting respondents' SC perceptions positively influenced the familiarity with the SC Guideline: healthcare's support needed in SC; own personal skills and knowledge adequate to support SC; perception that customers value NRT counseling; and perception that cigarette smoke is extremely detrimental to health (Table 2). Of the background variables related to respondent's working pharmacy, its location by province and dispensing counter design were associated with the Guideline familiarity (Table 2). All the variables measuring availability of SC services at the respondent's working pharmacy, e.g., use of SC pocket card, participation in local multidisciplinary SC actions, individually tailored SC service, and in-house guideline on NRT dispensing, were associated with the SC Guideline familiarity (Table 2).

In the multivariate analysis, nine out of the 21 variables were found to have a statistically significant association with the SC Guideline familiarity. The highest ORs were found in the variables related to own perception of the personal skills and knowledge in SC (OR 3.8); perception of customers value NRT counseling (OR 3.3); and regular use of the pocket card in SC counseling (OR 3.0; all variables, p ≤ 0.001) (Table 3).

### Guideline Familiarity and implementation of the SC Guideline-based actions

Implementation of the Guideline-based SC actions and services were more common among the respondents familiar with the Guideline (Table 4). The familiarity with the Guideline was strongly associated with respondents' working pharmacy's participation in local multidisciplinary SC actions, the frequency of applying the 5A's Intervention with smoking customers and the discussion about SC with customers who have higher risk factors or buy SC medicines. These actions were not so commonly taken compared to recommending NRT gum or patch, which were reported by almost all respondents despite their Guideline familiarity. Similarly, nearly all the respondents reported, that they always discuss about SC with customers who spontaneously refer to his/her smoking. The same applies to supporting smoker's own quitting decision which was done by more than 60% of the respondents.

## Discussion

This National study showed that approximately half (47%) of the responding pharmacists were familiar with the Finnish SC Guideline [7]. The Guideline familiarity was positively associated with the pharmacist's positive perception towards SC and SC actions taken in their workplace. On the other hand, no variables related to the characteristics of the workplace, such as pharmacy's size or ownership, geographical location or dispensing counter design influenced SC Guideline familiarity.

Healthcare professionals' unfamiliarity with the existing guidelines or their contents is considered a major barrier for successful guideline implementation [15,38]. According to a survey conducted among community pharmacists in Iowa in 2002, only 10% of the respondents were familiar with the US SC Guideline (Additional File 1, Table S1) [23]. In our study, approximately half of the pharmacists were familiar with the Finnish SC Guideline, i.e., they reported to know its main principles or have even better knowledge of it. This is very much in line with the survey of Ward et al. [14] reporting about half of the responding physicians in Australia being familiar with a National SC guideline. Interestingly, in that study [14], even more physicians self-reported that they followed the SC guideline's major recommendations than indicated being familiar with it. We had an opposite result in this respect with less respondents reporting implementation than familiarity with the Guideline. However, we found that the Guideline familiarity was associated with its implementation. These results illustrate the challenging nature of guideline implementation and the difficulty to assess the actual implementation, which has been widely studied [14-16,38,41]. The findings may also indicate differences between health professionals in bringing the SC guidelines into daily practice. It would be interesting to compare the SC guidelines implementation among different healthcare professionals with a sound methodology that allows reliable comparison.

In our study, the SC Guideline familiarity was more common among pharmacists who perceived that healthcare professionals are always needed in SC and were satisfied with their own personal SC skills and knowledge. Also the perception that customers value their NRT counseling positively influenced the Guideline familiarity. This is parallel to earlier findings of healthcare professionals' high personal motivation and positive attitude enhancing guideline implementation [6,16,38,39,42]. Correspondingly, studies conducted among community pharmacists found the association between good professional self-esteem and current SC practice (Additional File 1, Table S1) [8,23-26]. Healthcare professional's knowledge and SC perceptions can be enhanced by effective SC education and training, which should also focus on improving their-self esteem and understanding of their crucial role in SC [43,44]. According to a German survey among physicians, the association between SC training received and level of SC activeness might be linear [10].

Motivation to implement the SC Guideline may also be influenced by the economic value of SC services and the sale of SC medicines, as well as their reimbursement status [10,27]. In Finland, NRT products were released for general sale in 2006 and this has remarkably decreased the NRT sales in community pharmacies [45]. Despite this, community pharmacy owners' and staff pharmacists' commitment to SC was strong one year after the deregulation. It would be interesting to make a follow-up study to assess the development of the motivation in the long term and to follow the Guideline implementation rate.

Our findings suggest that the SC Guideline familiarity does not influence the recommendation of NRT products to smoking customers, which was clearly the most common SC action taken place among all the respondents. However, the familiarity was associated with the use of more sophisticated SC methods, such as recommending non-pharmacological methods, use of 5A's Intervention and participation in local multidisciplinary SC actions. Similarly, a survey among Australian physicians found a high rate of NRT recommendation, whereas behavioral or quitting advice or quit date setting were far rare [6]. Though the SC Guideline underlines the importance of local multidisciplinary collaboration, it proved to be a rare in our study. This finding is in accordance with earlier findings in Finland [46]. These findings suggest that there is a lot of work needed in order to achieve the key aim of the SC Guideline - to promote multidisciplinary care. It would be interesting to find out whether the situation is the same in other countries.

Our responses indicate that customers' degree of initiative significantly influences community pharmacists' SC counseling activity. This is in line with previous studies conducted among pharmacists (Additional File 1, Table S1) [23,24]. Community pharmacists' familiarity with the SC Guideline particularly influences their counseling activity with customers being extremely vulnerable for tobacco use (e.g. pregnant women and those who suffer from smoking-related diseases). These findings underline the importance of making the SC Guideline widely known among pharmacists and other healthcare professionals if they are required to provide sophisticated SC services in their community.

According to several previous studies, the working place has an important role in enhancing guideline familiarity and implementation [13,15,38,39]. In our study, SC services available in the workplace increased the familiarity with the Guideline, whereas characteristics of the workplace, such as pharmacy's size and geographical location did not influence on it. These findings suggest that all Finns may have equal access to SC services provided by community pharmacies regardless of where they life. This is important from a public health viewpoint, and opposite to the recent findings from the USA; the study conducted in the pharmacies of New York City area found better availability and lower prices of NRT products in the pharmacies of the wealthiest living areas [47].

Our nationwide study based on a representative sample (n = 1190) (Table 1) of Finnish community pharmacists had a relatively high response rate (54%) compared to other surveys conducted among community pharmacists [8,20]. There were no statistically significant differences between the respondent and target population with the following exception (Table 1). The pharmacists specialized in the treatment of asthma, diabetes or cardiovascular diseases, under the public health program of the AFP, responded more often than other pharmacists (see Methods, Context and study design, Table 1), this can be understood by their higher interest in SC and stronger professional role in SC. However, their proportion of the overall study respondents may overestimate the actual Guideline familiarity.

Respondents' familiarity with the SC Guideline was chosen to be our main outcome measure. We considered it to be more reliable for the respondents to assess their own familiarity with the Guideline than the actual level of implementation, which can be biased by limited ability to recall or by self-perceptions. In further studies, it might be useful to conduct an analysis using Guideline-based actions as the outcome variables and include Guideline familiarity alongside with other background variables to estimate the relative impact of Guideline familiarity on practice. However, the level of actual implementation can be more reliably assessed by population based intervention studies or pseudo patron studies.

Our survey was conducted in 2006 - 2007, i.e., about four years ago. Since then, tobacco control policy has changed in Finland supporting more smoke-free public areas, such as restaurants, workplaces and even municipalities. Also a new prescription medicine has been launched for SC in 2006. Despite these remarkable changes no follow-ups on effectiveness of SC or health care professionals practices has been recently conducted. It would be interesting to repeat this study to see whether any changes have taken place in this respect.

## Conclusions

Nearly half of the Finnish community pharmacists were familiar with the National Current Care Guideline in SC at the time of our survey. Pharmacists' Guideline familiarity can be enhanced by supporting their positive perceptions towards cessation, its importance and by offering continuing education. Further, the workplace (pharmacy) has an important role by providing Guideline-based SC services, and thus, support pharmacists' activeness in SC. Pharmacists' good knowledge and self-esteem towards SC alongside with SC supportive in-house practices at pharmacy are in crucial role while supporting the implementation. Among Guideline familiar pharmacists, the Guideline-based SC actions and services are better implemented than among respondents unfamiliar with the Guideline.

## Competing interests

*Terhi Kurko *has received personal grant for this study from the Finnish Cultural Foundation and funding from Pfizer Oy, Finland. *Kari Linden*, Ph.D., M.Sc., is employed by Pfizer Oy, Finland. He was earlier employed by the Association of Finnish Pharmacies where he contributed to the implementation of the National Current Care Guideline on Smoking Cessation among community pharmacists. *Kirsi Pietilä*, Ph.D., is a Pharmacy Owner and former Senior Lecturer in Social Pharmacy at the Faculty of Pharmacy, University of Helsinki, Finland. *Doctor Pietilä *belongs to the working group of the National SC Guideline. *Patrick Sandström *is employed by the National Institute for Health and Welfare (THL). He and Professor *Marja Airaksinen*, Ph.D., have no conflict of interests.

## Authors' contributions

All the authors meet the authorship criteria set by *BMC Public Health*. TK and KL conceptualized the study, did the statistical and other analysis, interpreted the findings and wrote the first draft. KP and PS contributed for the study design and for the development of the survey instrument. MA supervised all the steps of the research project and actively contributed to the preparation of this manuscript. All the authors have read and approved the final manuscript.

## Pre-publication history

The pre-publication history for this paper can be accessed here:

http://www.biomedcentral.com/1471-2458/10/444/prepub

## Supplementary Material

Additional file 1 **Table S1: Examples of studies showing community pharmacists' participation in SC**. This file contains an additional table, providing information of the studies of community pharmacists participation in SC. Click here for file

Additional file 2 **Table S2: The Community pharmacists' duties in the treatment chain of SC according to the National SC Guideline **[7]. This file contains an additional table proving information about the SC tasks the Guideline requires from community pharmacists. Click here for file
