# Supplementary material for: Community pharmacists' involvement in smoking cessation: familiarity and implementation of the National smoking cessation guideline in Finland
Source: BMC Public Health. 2010 Jul 29;10:444. doi: 10.1186/1471-2458-10-444 (PMC2922110; doi:10.1186/1471-2458-10-444)
Supplement: Additional file 1 — Table S1: Examples of studies showing community pharmacists' participation in SC. This file contains an additional table, providing information of the studies of community pharmacists participation in SC. [file 1471-2458-10-444-S1.DOC]

Additional File, Table S1: Examples of studies showing community pharmacists’ participation in SC

| **Study** | **Methods / setting** | **Aims of the study** | **Results** |
| --- | --- | --- | --- |
| Aquilino et al. 2003 [20] | Survey to Iowa community pharmacists, in January- February 2002, stratified sample (response rate 38.2%). | Smoking cessation services, self-perceptions of the SC role, barriers to SC. | 10% of the respondents were familiar with the AHCPR guideline of SC. 99% found it important for pharmacists to provide SC, but only 57% routinely advised customers to quit and only 11% systematically recognized smoking customers. Respondents did not found their own skills and knowledge as significant barriers to SC. Only 22% of the respondents had attended SC education during last three years.  The most important predictors of SC activeness were pharmacists’ professional status of staff pharmacist, full time working, recent participation in SC program and input on pharmacy’s tobacco sales and prescription volume of the pharmacy.  Lack of time was the most significant barrier to SC counseling. |
| Brewster et al. 2005 [24] | Survey to pharmacists in four Canadian provinces (response rate 72%, n=960), conducted in summer and fall 2002. | Tobacco and SC related education and knowledge, practices and their perceptions of their own role in SC. | The majority (>85%) of the respondents had good or excellent knowledge of SC medicines and the health risks of tobacco, but behavioral approaches were less familiar. 92.5% found as their duty to counsel NRT use whereas 68% perceived the use of 5A’s model and 62% behavioral counseling as their duty. Arranging follow-up was less familiar to them. Respondents supported NRT use and patients’ SC motivation most often, compared to less active asking or discussions of smoking.  A majority perceived their own knowledge, skills as very important facilitators of SC practice. Patients’ interests in SC were seen also seen important, whereas the factors related to the pharmacy were seen less important. |
| Goniewicz et al. (2010)  3 brief surveys in Poland [25] | Brief surveys to  1) Adult smokers (n= 329, rate 78%) in Southern Poland in winter 2007-2008.  2) Community pharmacists (n= 141; 100%) in regional section of Katowice in spring 2007.  3) Pharmacy students in Medical University of Silecia (n= 123; 100%) in spring 2007. | Adult smokers’ and community pharmacists’ perceptions of pharmacists’ role in SC.  The study also assessed pharmacy students’ perceptions on the SC education in their university. | Smokers’ survey: Of the respondents a majority had tried to quit earlier and 18% had earlier received SC counseling from a pharmacist. The majority found pharmacists as the right health care professional for SC medicine counseling and 56% found pharmacists qualified to support SC.  Pharmacists’ survey: A majority had patients asking help in SC weekly and 79% found themselves qualified in providing SC. 86% of them recommended NRT to patients at least often.  Students’ survey: 85% perceived to have satisfactory knowledge to provide basic information about the health consequences of smoking whereas only 61% were satisfied with their own knowledge to provide SC counseling. |
| Hudmon et al.  (2006): Survey [8] | 10 paged survey to licensed pharmacists in four Northern California counties during 1999 – 2000, (response rate 54.2%, n= 1168). | Self report of perceptions towards SC counseling, current practice and predictors for active SC counseling. | Only 8% or less of the participants had received SC education and a minority had followed the model of 5A’s / 4A’s in their practice. The rate of NRT counseling at pharmacies was low (20% of patients). SC was not routine task at the time of the survey.  Predictors of active SC counseling were found by a logistic regression model: pharmacists’ race, self-efficacy for counseling, practice setting and perceived pros for counseling. |
| Thananithisak et al. (2007)  Survey and interviews [26] | Survey in Bangkok to recently accreditation from the Thai Pharmacy Counsel received pharmacists (response rate 51%, n=83).  The in-depth interviews to 13 pharmacists with SC training. | SC activities by pharmacists  (self report).  The respondents were considered “early SC adopters”. | Survey: 60% had provided SC services and educational materials. A third had followed at least some parts of the 5A’s model. Nearly half had not participated in public SC campaigns. 93% perceived to have a crucial role in SC. They all agreed to the importance of pharmacists’ participation in multidisciplinary SC teamwork.  Interviews: Some had provided SC services including pharmaceutical and behavioral therapy.  In the survey and in the interview the most important barriers to SC were pharmacists’ low self-confidence, lack of knowledge, skills and guidance and of customers’ demand |
