# Supplementary material for: Community pharmacists' involvement in smoking cessation: familiarity and implementation of the National smoking cessation guideline in Finland
Source: BMC Public Health. 2010 Jul 29;10:444. doi: 10.1186/1471-2458-10-444 (PMC2922110; doi:10.1186/1471-2458-10-444)
Supplement: Additional file 2 — Table S2: The Community pharmacists' duties in the treatment chain of SC according to the National SC Guideline [7]. This file contains an additional table proving information about the SC tasks the Guideline requires from community pharmacists. [file 1471-2458-10-444-S2.DOC]

Additional File 2 Table S2: The Community pharmacists’ duties in the treatment chain of SC according to the National SC Guideline [7]

| **Pharmacies** | **The minimum duty required** | **The minimum level of information required** |
| --- | --- | --- |
| Staff Pharmacists (M.Sc. and B.Sc.) | Supports the rational use of NRT | Are familiar with the rational use of NRT |
|  | Takes care of rational NRT treatment | Are familiar with the 5A’s model |
|  | Offers also the non-pharmaceutical forms of SC support | Are familiar with the local SC services |
|  |  |  |
| Pharmacy owners | Participates in planning and implementation of the local treatment chain |  |
|  | Takes care of sufficient resources and education of pharmacists in SC |  |
